# A BIOPSYCHOSOCIAL-ECOLOGICAL, FAMILY-FRAMED APPROACH TO DEMENTIA CARE

**DOI:** 10.1093/geroni/igac059.1579

**Published:** 2022-12-20

**Authors:** Carol Podgorski

**Affiliations:** University of Rochester, Rochester, New York, United States

## Abstract

An individual’s experience of cognitive impairment is shaped by biopsychosocial factors, including their own perceptions of illness as well as interactions with family members, healthcare providers, and the communities in which they live. With advancing illness an individual’s dementia care requires the involvement and commitment of others, usually family. Hence, the quality of a person’s illness experience is shaped largely by relationships with family members and others throughout their respective communities. Current models of dementia care recognize family members as an important part of the care team, but fail to consider a patient’s family system and relationships as social determinants that affect care outcomes. This presentation will introduce a biopsychosocial-ecological, family-framed approach to dementia care that addresses factors that influence care considerations at both the individual and relational levels of the social ecological networks that the patient and their family members occupy.

